# Personal

**Published:** 1898-09

**Authors:** 


### PERSONAL.

Dr. Joseph McDowell Mathews, of Louisville, president of the American Medical Association, has resigned the chair of surgery, including the lectureship on diseases of the rectum, in the Kentucky School of Medicine. Professor Mathews has been appointed to a similar chair in the Hospital College of Medicine, where his colleagues will be more congenial and his field of usefulness will find an ampler range.

---

Dr. Frank Thornton Jenkins, of Buffalo, has opened offices at Niagara Falls, N. Y., where he has taken up his residence for the future. He limits his practice to the treatment of diseases of the nose, throat and ear, for which purpose he has fitted up a handsome suite of offices in the Gluck building, Niagara Falls.

---

Dr. Julius Ullman, of Buffalo, whose army appointment we have noticed in another column, has been assigned to duty at the Leiter general hospital, Chickamauga, Ga. Dr. Frederick Busch will attend at Dr. Ullman's office, 400 Franklin street, and upon his patients during his absence.

Dr. Nelson W. Wilson, of Buffalo, announces the removal of his office from No. 4 Brown Building to No. 155 Seventh street. Hours : 8 to 10 A. M. 1 to 3 and 7 P. M. Telephone, Tupper 169. Residence, No. 153 Seventh street.

---

Dr. Louise Downer, of Buffalo, has removed from 174 N. Pearl street to the sanitarium at Orchard Park, N. Y., of which she takes charge September 1, 1898.

---

Dr. W. Scott Renner and family, of Buffalo, have returned from a three months' tour in Europe. They were accompanied by Mr. and Mrs. J. W. Diehl.

